# Minimizing the impacts of the ammonia economy on the nitrogen cycle and climate

**DOI:** 10.1073/pnas.2311728120

**Published:** 2023-11-06

**Authors:** Matteo B. Bertagni, Robert H. Socolow, John Mark P. Martirez, Emily A. Carter, Chris Greig, Yiguang Ju, Tim Lieuwen, Michael E. Mueller, Sankaran Sundaresan, Rui Wang, Mark A. Zondlo, Amilcare Porporato

**Affiliations:** ^a^High Meadows Environmental Institute, Princeton University, Princeton, NJ 08544; ^b^Department of Civil and Environmental Engineering, Princeton University, Princeton, NJ 08544; ^c^Department of Mechanical and Aerospace Engineering, Princeton University, Princeton, NJ 08544; ^d^Applied Materials and Sustainability Sciences, Princeton Plasma Physics Laboratory, Princeton, NJ 08540; ^e^Andlinger Center for Energy and the Environment, Princeton University, Princeton, NJ 08544; ^f^School of Aerospace Engineering, Georgia Institute of Technology, Atlanta, GA 30332-0150; ^g^Department of Chemical and Biological Engineering, Princeton University, Princeton, NJ 08544

**Keywords:** low-carbon energy, ammonia, nitrogen cycle, nitrous oxide, leakages

## Abstract

Ammonia (NH_3_) is an attractive low-carbon fuel and hydrogen carrier. However, losses and inefficiencies across the value chain could result in reactive nitrogen emissions (NH_3_, NO_x_, and N_2_O), negatively impacting air quality, the environment, human health, and climate. A relatively robust ammonia economy (30 EJ/y) could perturb the global nitrogen cycle by up to 65 Mt/y with a 5% nitrogen loss rate, equivalent to 50% of the current global perturbation caused by fertilizers. Moreover, the emission rate of nitrous oxide (N_2_O), a potent greenhouse gas and ozone-depleting molecule, determines whether ammonia combustion has a greenhouse footprint comparable to renewable energy sources or higher than coal (100 to 1,400 gCO_2_e/kWh). The success of the ammonia economy hence hinges on adopting optimal practices and technologies that minimize reactive nitrogen emissions. We discuss how this constraint should be included in the ongoing broad engineering research to reduce environmental concerns and prevent the lock-in of high-leakage practices.

## Significance

The global transition to low-carbon energy necessitates exploring alternatives to fossil fuels. Hydrogen has emerged as a promising option; however, hydrogen storage and transportation challenges have led to considering ammonia as a hydrogen carrier and fuel. This study investigates the potential environmental risks associated with ammonia use in the energy sector. Our findings demonstrate that reactive nitrogen compounds released throughout the ammonia value chain can harm air quality, human health, ecosystems, and climate, and lead to stratospheric ozone depletion. However, we also show that optimal engineering practices and management strategies can effectively mitigate these concerns. Our research contributes to informed decision-making and the development of environmentally responsible ammonia energy systems.

Several low-carbon energy carriers are being explored as alternatives to fossil fuels to limit global warming. Among these, hydrogen (H_2_) has the largest potential to be the low-carbon fuel of the future due to the scalability of its production (1). Hydrogen can be obtained from different energy sources (fossil fuels, biomass, renewables, nuclear, etc.) through various technologies (reforming, gasification, pyrolysis, electrolysis, etc.). Using carbon capture and storage offers a path to decarbonize hydrogen production from fossil fuels. Stoichiometrically, hydrogen combustion produces only water as a byproduct, providing an opportunity to reduce CO_2_ emissions and air pollution (2). As a result of this potential, countries accounting for around 90% of the world’s energy supply and use have projects for large-scale H_2_ production (1, 3).

With the growth of the global H_2_ supply chain, a prominent international H_2_ trade, much of it seaborne, is expected to develop between renewable-rich areas (e.g., Australia, the Middle East, and North Africa) and demand centers (e.g., European Union, Japan, and South Korea) (4). However, direct transport of H_2_ is notoriously problematic because hydrogen has a very low energy density by volume at ambient temperatures and pressures. To get reasonable energy densities, H_2_ can be liquified at extremely low temperatures (<−253 °C) or compressed as a gas at very high pressures (300 to 700 bar) (5). Both such operations are technologically and energetically demanding and are prone to hydrogen leakages (6), with obvious drawbacks linked to economic losses, safety risks, and even climate impacts due to H_2_’s indirect greenhouse gas (GHG) effect (7–9).

Several transportation strategies are likely to compete for such long-distance transport (4, 10). Arguably the one most actively considered by the industry is transporting ammonia after converting hydrogen through the Haber–Bosch process (N_2_ + 3 H_2_ ⟶ 2 NH_3_) (5, 11). The energy required for the conversion would add only a small premium (~10%) on hydrogen production (11). Ammonia can be stored at much more reasonable conditions, e.g., as a liquid at −33 °C and standard pressure or at 10 bar and room temperature. A further advantage is that the ammonia transport and storage infrastructures have matured during the last century to deliver ammonia in agriculture and industry.

**Fig. 1.**
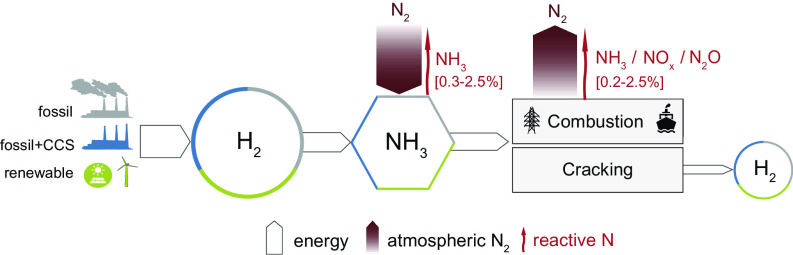
Schematic of the ammonia value chain and its potential impact on the nitrogen cycle. The white arrows track the energy flow starting from an input of primary energy converted to hydrogen and then to ammonia, which is either combusted or converted back to hydrogen through cracking. The wide brown arrows are the fluxes of atmospheric nitrogen, and the thin red arrows are the reactive nitrogen losses due to leaks and undesired reactions during combustion or cracking. Numbers in square brackets are estimated reactive nitrogen loss rates [minimum–maximum].

The transported ammonia can then either be burned to produce energy or it can be converted back to hydrogen through thermal or catalytic cracking—an energy-intensive step (11, 12) (Fig. 1). Conveniently, many gas turbines, furnaces, and internal combustion engines could be retrofitted for ammonia use (neat or blended with other fuels). In aggregate, these applications provide great promise for developing an ammonia economy (11, 13, 14). Pioneering projects will soon deliver the first ammonia-fueled vessels; Japan has a national strategy to use ammonia in power plants as well as in glass and steel manufacturing; and Saudi Arabia is building the first gigawatt (GW)-scale renewable ammonia plant (11). Moreover, many research projects are looking into efficient ways to obtain ammonia electrolytically, directly from water and atmospheric nitrogen, bypassing the Haber–Bosch process (13).

Developing an ammonia-based economy presents significant economic, societal, energy, and environmental challenges that only recently have started to be explored (10, 13, 15). A crucial question has remained overlooked: How will ammonia use in the energy sector impact the nitrogen cycle? Along these lines, only one recent paper has raised concerns about using ammonia fuel in the maritime sector (16). Here, we take this research further and analyze the potential emissions of reactive nitrogen compounds (NH_3_, NO_x_, and N_2_O) due to leaks and emissions during combustion or cracking processes. Drawing on available empirical evidence, we assess the potential emission rates, the perturbation of the nitrogen cycle, and the impacts of N_2_O emissions on climate and stratospheric ozone depletion. Our findings reveal that improper ammonia use can have disruptive environmental impacts, requiring careful scrutiny, but that optimal ammonia management can greatly reduce environmental concerns. We finally discuss how technological advances and mitigation strategies will be necessary to minimize environmental risks and prevent high-consequence outcomes. By thoroughly analyzing the environmental impacts associated with ammonia use, our paper aims to inform policymakers and industry leaders about the urgent need to address these challenges.

## Potential Impacts on the Nitrogen Cycle and Climate

### Nitrogen Cycle Perturbation.

**Fig. 2.**
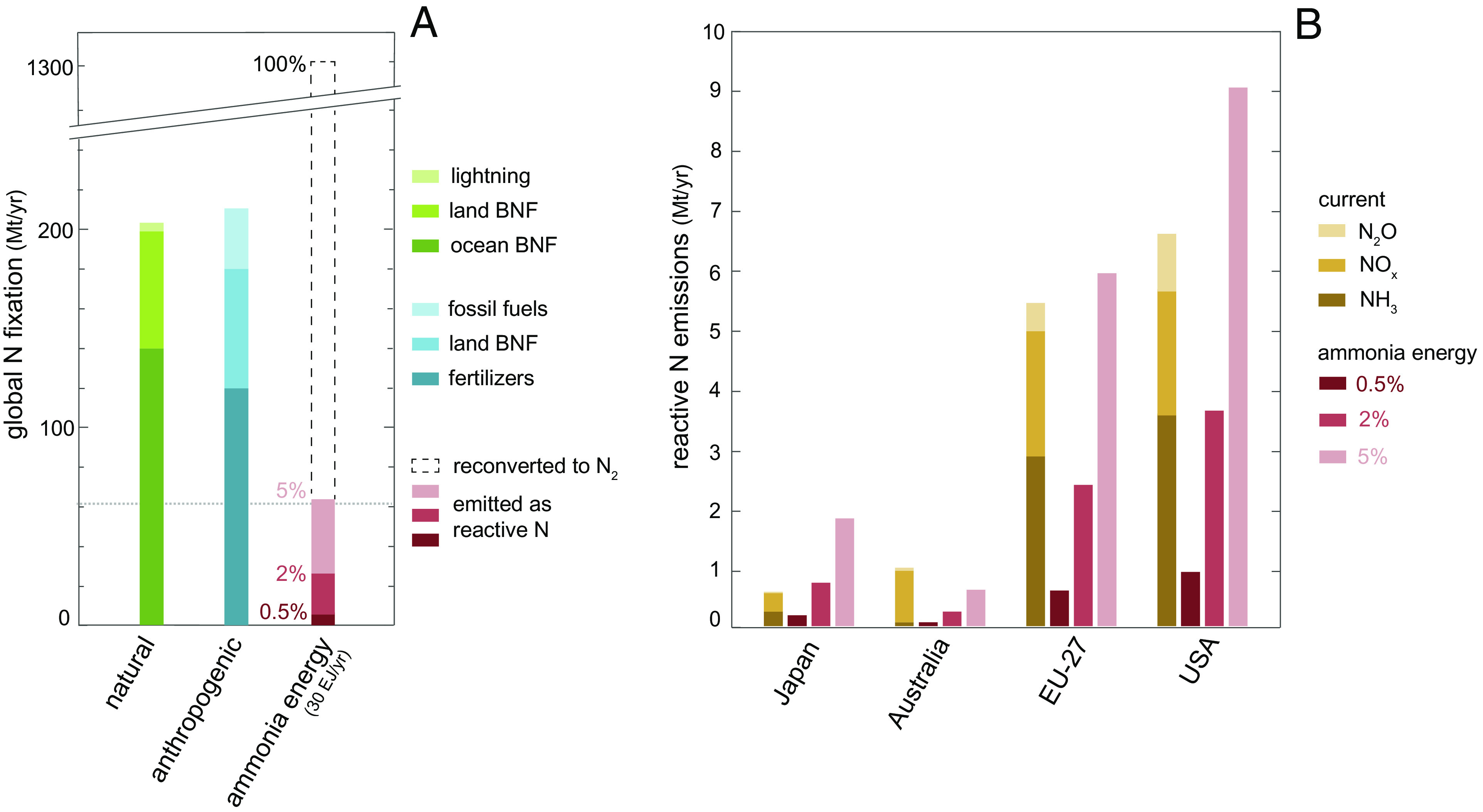
Potential impact of the ammonia economy on the N cycle. (*A*) Global N fixation by natural and anthropogenic fluxes (from ref. 18) and by the ammonia economy, which is assumed to be 30 EJ/y (~1,600 Mt NH_3_/y or ~1,300 Mt N/y). Only a fraction (0.5, 2, or 5%) of the nitrogen in ammonia is assumed to be emitted as reactive nitrogen. The dotted gray line is a widely cited safe planetary boundary for nitrogen (19, 20). BNF is biological nitrogen fixation. (*B*) Current reactive N emissions of some select countries and regions considering adopting ammonia fuel compared to the potential emissions of ammonia energy (assumed at 5% of current energy demand) for different loss rates (0.5, 2, or 5%). Current emission and energy data are for 2020 from ref. 21, and Japan’s NH3 emissions are for 2018 from ref. 22.

Although nitrogen is a key nutrient for all life forms, nature has evolved to thrive in a world where reactive nitrogen species (NH_3_, NO_x_, organic N, etc.) are scarce. Most of the nitrogen on Earth (>99.9%) is either buried in the lithosphere or present in the atmosphere in the diatomic form N_2_, which is relatively unreactive and can be naturally broken apart (viz., fixed) only by specialized microorganisms or lightning (17). Humans have significantly altered this equilibrium, mainly by producing nitrogen fertilizers through the Haber–Bosch process (~60% of global anthropogenic N fixation), and by cultivating nitrogen-fixing crops (~25%) and burning fossil fuels (~15%) (18). It is estimated that humankind fixes atmospheric nitrogen into reactive forms (~210 Mt N/y) at approximately the same rate as all the Earth’s natural systems combined (~200 Mt N/y) (18), see Fig. 2*A*. The proponents of a safe planetary boundary for nitrogen declare that it has already been crossed (19, 20).

While ammonia demand for energy represents a new and potentially large perturbation of the nitrogen cycle, there is a substantial difference between ammonia usage in the agriculture and energy sectors. In agriculture, all nitrogen fixed in ammonia-based fertilizers is released into the environment as reactive nitrogen, resulting in a one-way flow from N_2_ to reactive nitrogen. By contrast, most of the nitrogen in ammonia is converted back to atmospheric N_2_ during ammonia combustion (4 NH_3_ + 3 O_2_ ⟶ 2 N_2_ + 6 H_2_O) or cracking (2 NH_3_ ⟶ N_2_ + 3 H_2_), thus forming a closed cycle for nitrogen and significantly reducing environmental concerns. Practically, however, leakages across the ammonia value chain and undesired reactions during ammonia use would keep the nitrogen cycle partially open, releasing reactive nitrogen compounds (e.g., NH_3_, NO_x_, N_2_O, HONO) into the environment (Fig. 1). Anticipating later results, we estimate that up to 5% of the nitrogen in ammonia could be lost as reactive nitrogen compounds. The precise perturbation of the nitrogen cycle will depend on the amount of nitrogen fixed into ammonia and the average N loss rate.

Ammonia is already a global commodity, with a global production of ~180 Mt y^−1^ that makes ammonia the second-most-produced chemical by mass worldwide after sulfuric acid. Currently, 85% of this production goes to the agricultural sector as nitrogen fertilizers. With its emerging role in the global energy transition, the global ammonia economy could expand significantly. We estimate that a decade or two after 2050, ammonia energy will have reached 30 EJ/y, or 1,600 Mt NH_3_/y. The conversion from energy units to tons is based on 19 GJ/t, ammonia’s lower heating value (LHV). Our estimate is based on reasonable hypotheses to guide the discussion: the primary energy economy will be 1,000 EJ/y (about twice its size today), hydrogen as a secondary energy source (energy carrier) will become 15% of primary energy (150 EJ/y), and 20% of this hydrogen will be transported as ammonia. An ammonia production of 1,600 Mt NH_3_/y is an order of magnitude larger than the current production of the fertilizer industry. Another comparison is with the shipment rate of ammonia today, which is around 20 Mt of ammonia per year (11). In an ammonia economy of 1,600 Mt NH_3_/y, the seaborne fleet would need to expand its current transport capacity by up to eighty times.

The amount of reactive nitrogen escaping to the natural environment will depend dramatically on ammonia housekeeping and use details. Given the uncertainty about a value chain that still needs to be built, we explore a range of emission rates for ammonia leakages and emissions during incomplete combustion, bounded by optimal and suboptimal technological practices. For ammonia leakages, we draw an analogy with the methane leakage rates from natural gas supply chains quantified by airborne and satellite measurements. Ammonia and methane share similar gas diffusivities at the same pressure because of their similar molecular mass, but it is likely that ammonia will be stored at much lower pressures (e.g., 10 bar) than methane (e.g., 250 bar). We use 0.3% as the lower bound, equivalent to the methane leakage rate of Norway (23, 24). For the upper bound, we use the leakage rate from the US natural gas supply chain, which is around 2.5% (25, 26). We exclude higher leakage rates of other countries (24) because, unlike methane, ammonia will not be extracted from leaky fields, and its toxicity will require a supply chain built with greater integrity. Regarding combustion emissions, we assume that between 0.2 and 2.5% of nitrogen may be emitted as reactive nitrogen compounds (NH_3_, NO_x_, or N_2_O), depending on optimal vs. suboptimal combustion conditions. These assumptions are further discussed later in the paper and supported with calculations in *Materials and Methods*.

Accounting for leakages and combustion emissions, between ~0.5 and 5% of the nitrogen drawn from the atmosphere for ammonia production could be lost to the environment as reactive nitrogen. With an ammonia production of 1,600 Mt NH_3_/y, roughly equivalent to 1,300 Mt N/y, this loss rate would perturb the global nitrogen cycle by 6.5 to 65 Mt N/y (Fig. 2*A*). The upper bound is very large, about half of the current global perturbation due to fertilizers. At the country or regional level, even a small penetration (e.g., 5%) of ammonia in the energy market could lead to reactive nitrogen emissions that are comparable to the current cumulative emissions from agriculture, industry, and energy sectors (Fig. 2*B*). In addition to air pollution (27–29), these emissions would also lead to water pollution after deposition, regional alterations of ecosystems (17, 18, 30), and global warming and stratospheric ozone depletion via nitrous oxide (31). *SI Appendix* (*SI Appendix*, section S1 and Fig. S1) provides a review of these well-known environmental impacts.

### Emissions of Nitrous Oxide (N_2_O).

A stumbling block to the efficacy of ammonia as a climate-change mitigation solution is the potential emissions of nitrous oxide (N_2_O). This is a potent and long-lived (~120 y) GHG with a global warming potential (GWP) of 265, meaning that it is 265 times more powerful than CO_2_ in absorbing outgoing infrared radiation on a mass basis. N_2_O emissions are also the most critical ozone-depleting emissions (32) now that global efforts (Montreal Protocol) have reduced the emissions of other, more powerful, ozone-depleting gases containing fluorine, chlorine, and bromine.

The primary source of N_2_O emissions in the ammonia economy would be unwanted reactions (e.g., NO + NH → N_2_O + H) during ammonia combustion (33). While elevated N_2_O levels may exist in the combustion zone, N_2_O is primarily an intermediate species. Final N_2_O levels from high-temperature combustion are typically very low, resulting in an assumed lower-bound emission rate of essentially zero. However, like unburned hydrocarbons or carbon monoxide (CO) in the case of hydrocarbon combustion, N_2_O levels are susceptible to local quenching (33). This typically occurs at points of low flame temperature, flame impingement on walls, or where air streams are introduced for wall-cooling purposes. While combustion technologists possess ample experience designing systems to minimize quenching effects, they could persist at off-design conditions (e.g., during startup or at low power conditions) or may require tradeoffs between N_2_O emissions and other system performance metrics, such as overall combustor length (influencing capital cost) or life. If 2% of ammonia avoids high-temperature combustion and with a 50% conversion of ammonia to N_2_O (33), up to 1% of the nitrogen could be lost as N_2_O, equivalent to the production of 22 g of N_2_O (half a mole) from 1,700 g of NH_3_ (100 moles).

**Fig. 3.**
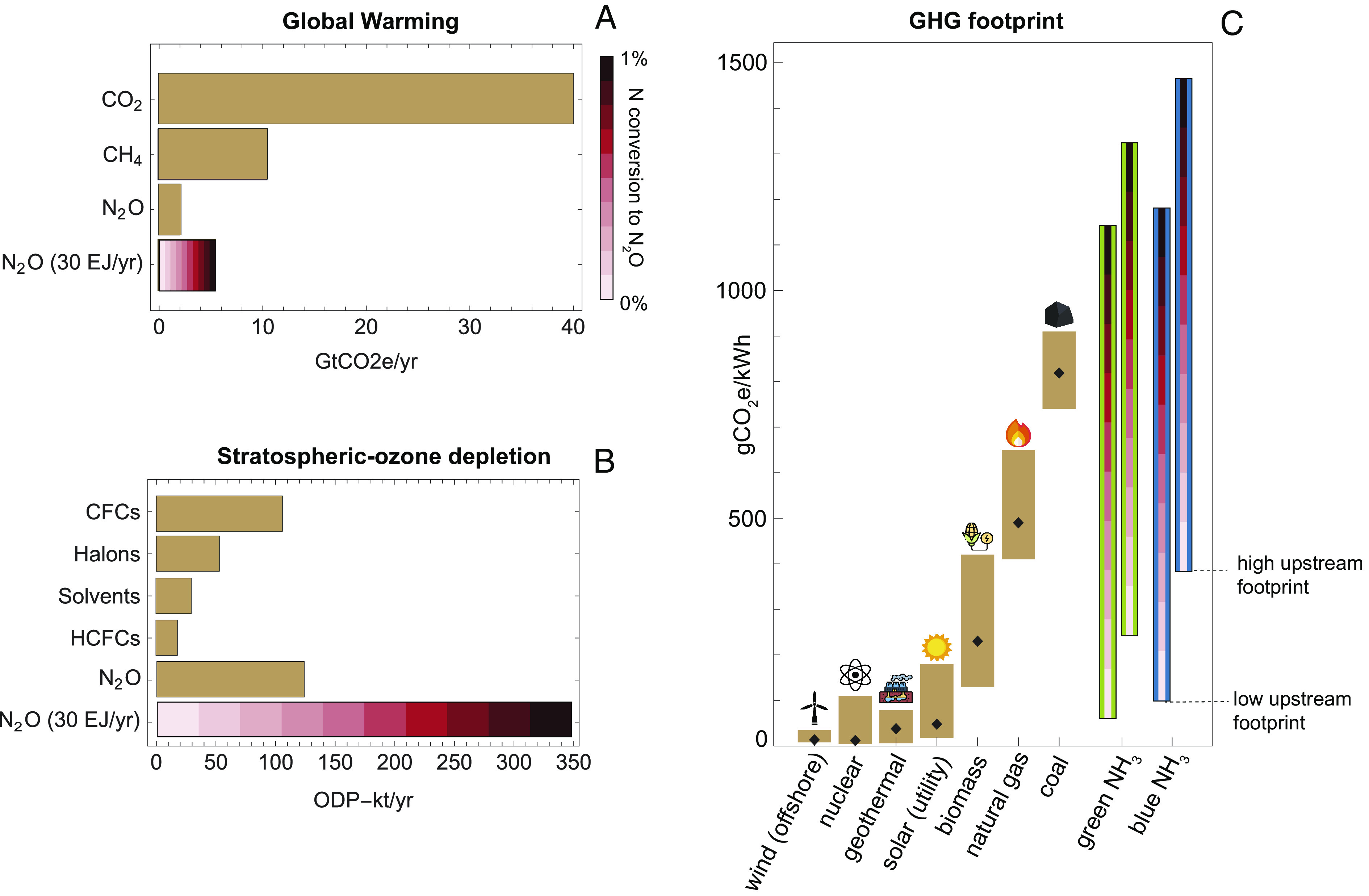
Global impacts of potential N_2_O emissions from ammonia energy. Loss rates as N_2_O are from 0 to 1%, with each bar color representing a 0.1% increase. (*A*) Global warming potential and (*B*) ozone-depletion potential (ODP) of N_2_O emissions in an ammonia economy of 30 EJ/y compared to current emissions of other gases. The emissions of ozone-depleting gases for the year 2016 are derived from the World Meteorological Organization (WMO) report (34) and are converted to CFC-11 equivalents using their ODPs. (*C*) GHG footprint of ammonia compared to other sources for electricity generation. Upstream emissions of ammonia production are taken from the IRENA report (11), with blue ammonia derived from a range of fossil fuels with carbon capture and storage. Values for the other energy sources are from the IPCC report 2018, with black diamonds standing for median values (35). Energy icons are from https://www.Flaticon.com.

With a 1% nitrogen conversion into N_2_O, an ammonia economy of 1,600 Mt NH_3_/y would result in 20 Mt N_2_O/y, around three times current anthropogenic emissions (31). With a GWP of 265, 20 Mt N_2_O/y is equivalent to 6 Gt CO_2_eq/y, about 15% of the global greenhouse emissions rate per year (Fig. 3*A*). The GHG intensity of such an ammonia economy (0.2 GtCO_2_e/EJ) is about twice as high as the current fossil fuel economy (~0.1 GtCO_2_e/EJ), even without considering all upstream emissions related to ammonia production. The ammonia economy would have the same climate impact as the fossil-fuel energy system in the case of a 0.4% nitrogen conversion from NH_3_ to N_2_O. The same critical rate has recently been obtained in the specific analysis of shipping emissions (16). Once in the stratosphere, ultraviolet radiation activates N_2_O, forming NO_x_ as byproducts that deplete stratospheric ozone. The ozone-depletion potential (ODP) of N_2_O is 0.017, meaning that a unit mass of N_2_O destroys 0.017 times the amount of stratospheric ozone destroyed by releasing a unit mass of chlorofluorocarbon 11 (CFC-11) (34). The ammonia economy with 20 Mt of N_2_O emissions per year could hence add another 340 ODP-kt/y to the stratosphere, potentially becoming the most prominent cause of stratospheric ozone depletion (Fig. 3*B*).

N_2_O emissions dramatically impact the GHG intensity of electric power from ammonia combustion. Burning 1 kg of NH_3_ produces 19 MJ of thermal energy (LHV) or around 3.2 kWh of electricity with a 60% conversion efficiency. It would also produce around 13 g of N_2_O with a 1% N_2_O loss rate, or 3.4 kg CO_2_e/kg NH_3_. The GHG intensity of such electricity would be around 1,100 gCO_2_eq/kWh, higher than coal. The consequences of N_2_O formation in ammonia combustion can be seen in Fig. 3*C*, where we compare the GHG footprint of ammonia with other energy resources. Even without N_2_O emissions, no scenario for low-carbon ammonia has a GHG footprint as small as that of wind or geothermal; the green ammonia footprint is comparable to the footprint of solar or biomass. However, as N_2_O emissions climb (by tenths from 0 to 1% in Fig. 3*C*), green and blue ammonia combustion dominates every other power source, including coal.

Secondary sources of N_2_O emissions would come from the oxidation of ammonia leakages in the atmosphere (36) and the biotic conversion of nitrogen in soils following deposition (37, 38). Global climate models estimate that around 1% of the nitrogen in ammonia can be converted into N_2_O following ammonia reaction with the atmospheric OH radical (36). The IPCC uses the same emission factor (1%) to estimate the fraction of nitrogen converted into N_2_O in soils, although estimates are highly variable depending on environmental conditions (0.1 to 15%; 37, 38). Accounting for these processes, our high estimate of reactive nitrogen emissions (65 Mt N/y) would cause additional N_2_O emissions of around 1 Mt N_2_O/y. These emissions are comparable to the current N_2_O emissions from fossil fuel and industry sectors (31), but are negligible compared to the potential ammonia combustion emissions.

## Losses Across the Ammonia Value Chain

### Ammonia Leakages.

**Fig. 4.**
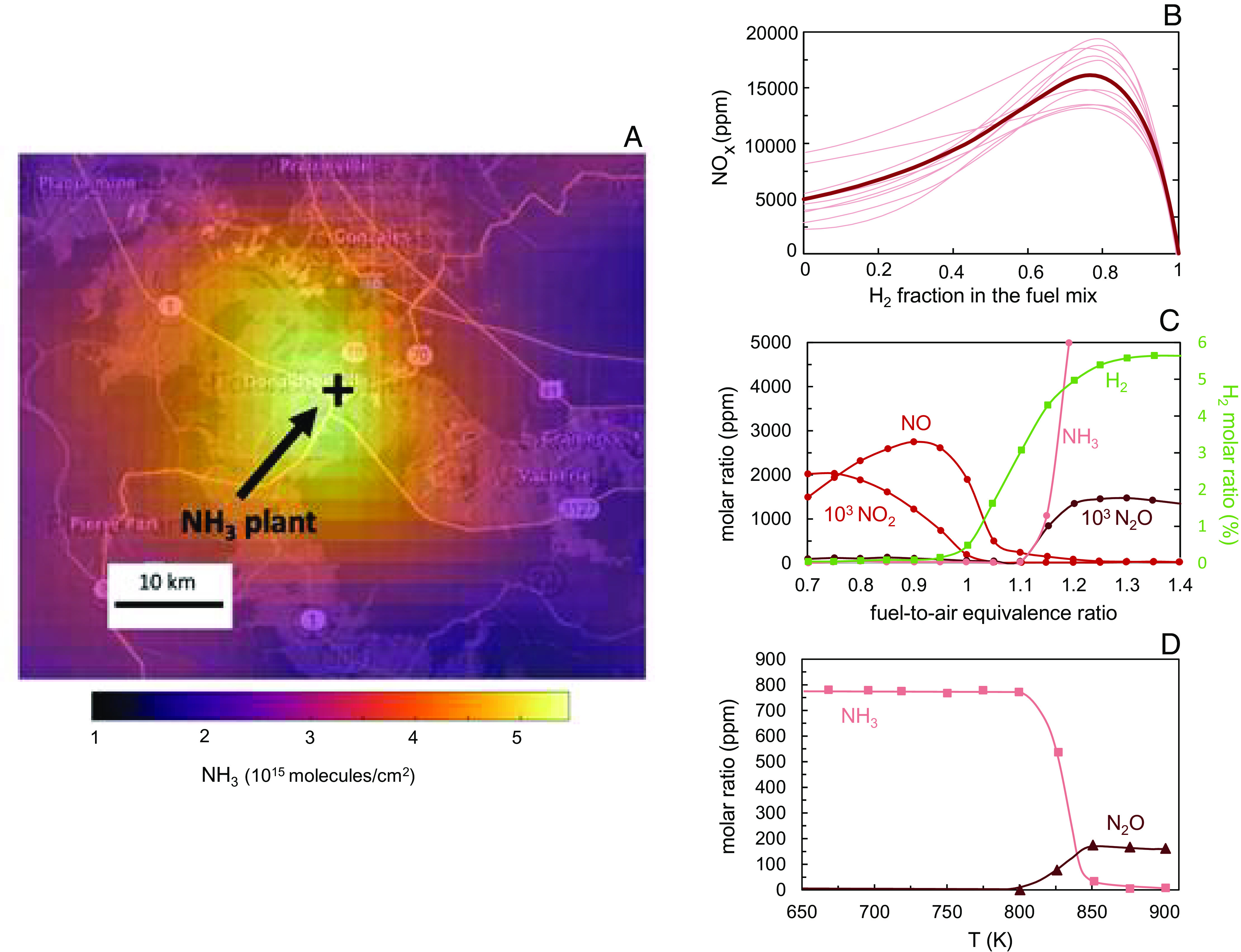
Reactive nitrogen losses across the ammonia value chain. (*A*) Satellites reveal ammonia leaks from the largest (≈4 Mt/y) production facility in the United States (Donaldsonville, Louisiana). Technical details are in *Materials and Methods*. (*B*–*D*) Potential emissions during combustion. Figures adapted from plots in refs. 33, 42, and 43. (*B*) Simulated NO_x_ emissions versus H_2_ mole fraction in the H_2_–NH_3_ fuel mix for stoichiometric mixtures. The thin pink lines come from the 10 references collected in ref. 43. The thick red line represents the average. (*C*) Simulated nitrogen emissions for pure ammonia combustion as a function of the fuel-to-air equivalence ratio (i.e., fuel lean when <1, fuel rich when >1) (42). (*D*) Molar ratios of NH_3_ and N_2_O as a function of the reactor temperature in an experiment of ammonia oxidation in a quartz tube at relatively low temperatures and high pressure (100 bar) (33). N_2_O concentration is expected to decrease at higher temperatures (>1,100 K) when NO_x_ formation is favored.

Even though the ammonia infrastructure has a high level of maturity, and many regulations to mitigate ammonia risks have been established worldwide, satellite observations reveal that industrial NH_3_ production plants are hotspots of ammonia emissions, which are greatly underestimated in inventories by a median factor of 50 (39). As a case in point, Fig. 4*A* shows a strong ammonia plume emitted by the largest ammonia production plant in the United States (technical details for satellite observations are provided in the *Materials and Methods*). In the ammonia economy, emissions from pipelines, distribution and storage systems, fuel stations, and combustion and cracking sources may also occur. Satellites are a promising tool for monitoring large ammonia leakages, but enforcing leakage minimization will require new regulations for ammonia emissions. On the one hand, the regulatory regime now common worldwide to control the emissions of NO_x_ provides an optimistic perspective, even though satellites play no part. Anthropogenic NO_x_ emissions in the United States and Europe have fallen by almost 70% since 1990 (40, 41). On the other hand, methane emissions from the natural gas supply chain offer a cautionary tale. Even with the increased focus on leak detection and repair over the past decade and economic incentives to reduce leakage, estimated methane leakage from the natural gas supply chain remains high (e.g., around 2.5% of US gas production (25, 26).

While ammonia satellite detection is a promising tool, it currently suffers from a series of technological limitations that will need to be addressed. First, satellite ammonia detection is challenging because ammonia’s atmospheric lifetime is very short (~hours), and atmospheric concentrations are typically at low levels (part per billion, ppb), comparable to the satellite detection limit. Satellite ammonia detection becomes even more challenging when measurements are subject to higher uncertainties under low-temperature conditions. Moreover, the resolution of current satellites for ammonia detection is coarse compared to other atmospheric pollutants. The Infrared Atmospheric Sounding Interferometer (IASI) pixel for ammonia observation is 12 km in diameter at the nadir view (44). By contrast, the Tropospheric Monitoring Instrument (TROPOMI) pixel for methane is 3.5 × 7 km^2^ (45). Hopefully, some of these issues will be addressed by the future-generation ammonia instrument (Nitrosat; 46) that will be launched in 2032 with a spatial resolution of 500 × 500 m^2^. Last, there has been limited work devoted to the in situ validation of ammonia satellite observation over space and time, mostly because there is a lack of in situ ammonia datasets to validate against (47). The validations of satellite NH_3_ retrievals have only been conducted with airborne and ground observations in regions with high ammonia concentrations, like the San Joaquin Valley in California (47, 48).

### Combustion Emissions.

NH_3_, NO_x_, and N_2_O emissions will also emerge from reactions during ammonia combustion or cracking. Ammonia combustion is a rapidly emerging technology that faces challenges due to elevated reactive nitrogen emissions and ammonia’s poor combustion properties (49). The elevated reactive nitrogen emissions occur from the presence of reactive nitrogen in the fuel (NH_3_), which can be converted to NO_x_ and N_2_O through a broad range of kinetic pathways, even at relatively low temperatures (e.g., 900 K). Conversely, the combustion of fuels without molecularly bound nitrogen (e.g., hydrocarbon and hydrogen) forms reactive nitrogen compounds only at temperatures sufficiently high (>1,800 K) to break the atmospheric N_2_ triple bond (50).

Regarding the combustion properties relative to other common fuels, ammonia has much poorer ignitability, lower flame speed, and narrower flammability limits (*SI Appendix*, Table S1). Blending ammonia with hydrogen obtained by partially cracking the ammonia before the combustion process improves these properties (43, 51), and the combustion waste heat can conveniently be used to promote the partial ammonia cracking (52). However, care must be exercised in the combustion approach, as the higher adiabatic flame temperature of stoichiometric hydrogen–ammonia combustion can lead to higher NO_x_ compared to pure ammonia combustion (Fig. 4*B*) (43). Another way to increase the flame temperature and improve ammonia’s combustion properties is to enrich the oxidizer with oxygen by removing some of the nitrogen from the air. However, the competing thermal and chemical effects on reactive nitrogen emissions have yet to be quantified.

The magnitude of reactive nitrogen emissions in pure ammonia combustion is highly dependent on combustion strategy, including flame temperature, combustor design, fuel and air mixing, and global fuel-to-air equivalence ratio (Fig. 4*C*). With the standard design of hydrocarbon/hydrogen-fueled systems (i.e., premixed, lean combustion), ammonia combustion leads to NO levels as high as 5,000 ppm (42, 53–55), corresponding to approximately 2.5% of the nitrogen in ammonia fuel (see *Materials and Methods* and *SI Appendix*, Fig. S2 for conversion of mixing ratios to loss rates). N_2_O levels with the same combustion strategy are estimated to be relatively low (~1 ppm; 42), although these calculations do not account for local quenching and poor fuel/air mixing, which significantly impacts N_2_O formation at low combustion temperatures, as shown in Fig. 4*D* and discussed in the N_2_O emission section above. Lower NO_x_ emissions can be achieved with two-stage combustion, with a fuel-rich zone first, followed by additional air injection and a secondary fuel-lean zone, where the fuel is primarily the hydrogen obtained from ammonia decomposition in the first zone. Recent experiments with this type of combustion, so-called Rich-Quick Quench-Lean (RQL) (56), have measured NO_x_ levels around 300 ppm (57), and numerical calculations suggest that lower levels are achievable (e.g., 50 ppm; 58). While these levels correspond to a relatively low nitrogen loss rate (around 0.15% for 300 ppm), they are still above most of the current NO_x_ regulation limits and far above the emission levels of methane-fueled advanced low-emission gas turbine engines (~3 to 30 ppm, depending on flame temperature; 59).

If advanced combustion strategies fail to achieve reasonable emission levels, post-combustion mitigation solutions such as scrubbers and Selective Catalytic Reduction (SCR) will be necessary (see refs. 60 and 61) for extended reviews of these technologies). An advantage of ammonia combustion is the ready availability of ammonia to use in SCR systems, wherein ammonia reacts with NO_x_ or N_2_O over a catalyst to produce nitrogen and water (e.g., 4 NH_3_ + 4 NO + O_2_ → 4 N_2_ + 6 H_2_O). A drawback of SCR systems is that they can increase the amount of unreactive ammonia emitted to the atmosphere (viz., ammonia slip). For example, an SCR system with a NO conversion efficiency of 90% (62) and stoichiometric ammonia input could reduce NO emissions from 300 to 30 ppm, but increase ammonia slip by 30 ppm. Other promising postcombustion mitigation strategies at the research level include electron beam irradiation and electrochemical reduction (60).

### Cracking Emissions.

As an alternative to combustion, ammonia can be reconverted to hydrogen and nitrogen (2 NH_3_⟶ 3H_2_ + N_2_) through thermal or catalytic cracking. This is currently an expensive and energy-intensive process (30 to 60% energy efficient; 52) that usually involves passing ammonia at high temperatures (>500 °C) over a catalyst material, like nickel supported on an alumina support in commercially available technologies (52). Ruthenium is known to perform better, but its higher cost and larger GHG footprint make it less favorable (52). Ongoing research is currently devoted to lowering operating temperatures and costs and enhancing conversion efficiencies, including material solutions for the catalyst and alternative energy input pathways (52, 63, 64). Many promising, less expensive, cobalt-, iron-, nickel-based catalysts, including bi- and multi-metallics, are concurrently being evaluated (52). Forward-looking ideas for energy input for cracking include light-driven plasmonic photocatalysis (65, 66) and the use of (cyclic) electrically pulsed heating and cooling, which has been demonstrated thus far for ammonia synthesis (67).

Ammonia conversion efficiency in current cracking technologies varies significantly, ranging from 10% to 99.9% in more advanced configurations (52). Configurations with low conversion rates produce hydrogen-ammonia blends suitable for combustion. In contrast, high-efficiency conversion is necessary for using hydrogen in applications such as proton-exchange membrane (PEM) fuel cells vulnerable to corrosion from residual ammonia levels as low as 0.1 ppm (68). Because research on cracking technologies has focused primarily on improving energy and chemical efficiencies, less attention has been paid to quantifying potential emissions of reactive nitrogen compounds during the cracking process. Ammonia slip is likely, and its intensity will depend on the reactor configuration. The formation of NO_x_ and N_2_O should instead be low since cracking would be performed mostly in the absence of oxygen. This may offer an environmental advantage to cracking over ammonia combustion if issues about energy requirements and cost can be addressed. Regardless, after cracking, any unconverted ammonia is ultimately used in fuel cells or burned alongside hydrogen, where it can contribute to NO_x_ or N_2_O emissions (*vide supra*).

## Outlook and Conclusions

The criticalities outlined in this work show that further research will be crucial to define the best pathway for ammonia use in the energy sector (e.g., combustion vs. cracking) and to improve the processes involved in the ammonia value chain, including increasing overall chemical and energy efficiencies and reducing reactive nitrogen emissions. Reactive nitrogen management will be even more important if technological advances provide breakthroughs for ammonia energy, e.g., if electrochemical ammonia production from water and atmospheric nitrogen becomes efficient and scalable (13, 69). In such a case, the ammonia economy could grow more than assumed in this work, beyond a mere subset of the hydrogen economy. With widespread plans for ammonia adoption, imposing environmental constraints on the choice of technology and advancing mitigation solutions at the technological and policy levels will be imperative. From an engineering perspective, many technologies at the research forefront, currently at low technological readiness levels and not yet demonstrated at scale, could alleviate some of these environmental concerns. For example, low-volatile forms of ammonia for storage could reduce leakage risks. A brief overview of these technologies, including alternative pathways for ammonia’s synthesis and stable storage, is provided in *SI Appendix*, section S2. From a policy perspective, low emissions can be incentivized via taxes, cap and trade, or subsidies, as with other emissions. While a regime for NO_x_ emissions is already in place in most of the world (17, 40, 41), new regulations will be required for ammonia and nitrous oxide, whose emissions so far have been dominated by the agricultural sector.

In conclusion, although there is a limiting case where all nitrogen removed from the atmosphere for ammonia production goes through a closed cycle (N_2_⟶NH_3_⟶N_2_), in the real-world ammonia value chain, there will inevitably be reactive nitrogen losses (NH_3_, NO_x_, and N_2_O). These losses may significantly perturb the nitrogen cycle, impacting air and water quality, human health, ecosystem services, stratospheric ozone, and climate (17, 18, 30). The extent of the potential global perturbation depends on future ammonia demand and reactive nitrogen loss rate. Our results suggest a large variability in the outcome, ranging from little to disruptive environmental impact, depending on the pathway of use and the technological practices adopted. While companies are already interested in minimizing losses for safety and economic reasons, there is an immediate need to understand the technological and economic trade-offs that determine loss rates and reduce them below critical levels. The ammonia economy can reduce our impact on the carbon cycle, but it must neither increase our impact on the nitrogen cycle nor exacerbate anthropogenic GHG emissions. Only a coordinated effort from the scientific community, the energy sector, and governments can minimize this trade-off.

## Materials and Methods

### Loss Rates of Incomplete Ammonia Combustion.

We use here available experimental and numerical evidence to estimate the possible loss rates (*L* in %) of reactive nitrogen species (NO, NO_2_, N_2_O, NH_3_) due to incomplete ammonia combustion

[1] $$L_{\mathrm{comb}}=L_{{\mathrm{NH}}_{3}}+L_{\mathrm{NO}}+L_{{\mathrm{NO}}_{2}}+L_{N_{2}O}.$$

To the authors’ knowledge, these percentages are not directly available in the literature, which usually provides molar ratios of reactive nitrogen compounds in the exhaust (e.g., [NO] in ppm). Therefore, we provide a methodology to quantitatively link the molar ratio measurements (ppm) to the loss rates (%). We assume that the source of reactive nitrogen emissions is the fuel (NH_3_) and not the nitrogen (N_2_) in the combustion air, which has a triple chemical bond that requires much more energy to break apart. Taking NO as an example, the loss rate of NO is

[2] $$L_{\mathrm{NO}}=\frac{{\dot{m}}_{\mathrm{NO}}}{{\dot{m}}_{\mathrm{fuel}}},$$

where ${\dot{m}}_{\mathrm{NO}}$ is the molar flux of NO in the exhaust and ${\dot{m}}_{\mathrm{fuel}}$ is the input molar flux of NH_3_. The molar flux of NO is linked to the NO molar ratio, [NO],

[3] $${\dot{m}}_{\mathrm{NO}}=\left[\mathrm{NO}\right]{\dot{m}}_{\mathrm{ex}}=\frac{\left[\mathrm{NO}\right]{\dot{M}}_{\mathrm{ex}}}{\rho_{\mathrm{ex}}},$$

where ${\dot{m}}_{\mathrm{ex}}$ and ${\dot{M}}_{\mathrm{ex}}$ are the molar and mass fluxes of the exhaust, respectively, and $\rho_{\mathrm{ex}}$ is the average molecular density of the exhaust. By mass conservation, the mass flux of the exhaust is equal to the mass flux of the input (air and fuel)

[4] $${\dot{M}}_{\mathrm{ex}}={\dot{M}}_{\mathrm{air}}+{\dot{M}}_{\mathrm{fuel}}=\rho_{\mathrm{air}}{\dot{m}}_{\mathrm{air}}+\rho_{{\mathrm{NH}}_{3}}{\dot{m}}_{\mathrm{fuel}}.$$

The influx of fuel and air are related through the equivalence ratio φ, namely the ratio of the actual fuel/air ratio to the stoichiometric fuel/air ratio (st),

[5] $$\varphi =\frac{\left({\dot{m}}_{\mathrm{fuel}}/{\dot{m}}_{\mathrm{air}}\right)}{\mathrm{st}},$$

where st≈0.28 comes from stoichiometry (4 NH_3_ + 3 O_2_ ⟶ 2 N_2_ + 6 H_2_O) and the fact that air is composed of 21% O_2_. Substituting Eqs. **3**– **5** into [2] and assuming that the exhaust, mostly composed of N_2_ and H_2_O, has a similar molecular density to the air ($\rho_{\mathrm{ex}}\approx \rho_{\mathrm{air}}$) yields

[6] $$L_{\mathrm{NO}}=\left[\mathrm{NO}\right]\left(\frac{1}{\mathrm{st}\;\varphi }+\frac{\rho_{{\mathrm{NH}}_{3}}}{\rho_{\mathrm{air}}}\right).$$

The same equations apply to the other reactive nitrogen species, namely

[7] $$\begin{array}{c} L_{{\mathrm{NO}}_{2}}=\left[{\mathrm{NO}}_{2}\right]\left(\frac{1}{\mathrm{st}\varphi }+\frac{\rho_{{\mathrm{NH}}_{3}}}{\rho_{\mathrm{air}}}\right),\;\\ L_{{\mathrm{NH}}_{3}}=\left[{\mathrm{NH}}_{3}\right]\left(\frac{1}{\mathrm{st}\;\varphi }+\frac{\rho_{{\mathrm{NH}}_{3}}}{\rho_{\mathrm{air}}}\right),\;\\ L_{N_{2}O}=2\left[N_{2}O\right]\left(\frac{1}{\mathrm{st}\;\varphi }+\frac{\rho_{{\mathrm{NH}}_{3}}}{\rho_{\mathrm{air}}}\right), \end{array}$$

where the factor of 2 in the N_2_O equation is due to the presence of two N atoms in the N_2_O molecule. Eqs. **6** and **7** can be used to estimate the loss rate (%) of NH_3_ to the various reactive nitrogen species from the equivalence ratio and numerical and experimental values of the mixing ratios in the exhaust. For stochiometric mixtures (*φ* = 1), Eq. **6** becomes $\begin{array}{c} L_{\mathrm{NO}}\;=\;(1/0.28\;+\;17/29)[\mathrm{NO}]\;\approx \;4.2[\mathrm{NO}] \end{array}$. This and Eqs. **6** and **7** for different *φ* values are graphed in *SI Appendix*, Fig. S2.

In fuel-lean conditions (*φ* < 1), pure ammonia combustion promotes the formation of NO_x_ via NH_2_, HNO, H_2_NO, N_2_O, and NNH pathways (42). NO formation maximizes at high temperatures and *φ* ≈ 0.9, with NO concentrations in the exhaust as high as 5,000 ppm (42, 53–55). This mixing ratio corresponds to a 2.3% NO loss rate through Eq. **6**. Loss rates for NH_3_ and NO_2_ in fuel-lean conditions are usually negligible. NO formation decreases dramatically in fuel-rich conditions (*φ* > 1) but with a clear trade-off in unburnt ammonia (e.g., 6,000 ppm of NH_3_ in the exhaust at φ = 1.2 (42), corresponding to $L_{{\mathrm{NH}}_{3}}\sim 2.1\%$ through Eq. **7**). Regarding N_2_O, emissions from fuel-lean high-temperature combustion are usually very low but reported values do not generally account for local quenching, a process to which N_2_O formation is highly sensitive—as discussed in the N_2_O emission section. For the maximum loss rate of reactive nitrogen species, we adopt the NO loss rate of 2.3%, rounded up to 2.5%, to account conservatively for uncertainties.

### Ammonia Satellite Observations.

This study uses NH_3_ retrievals from the infrared atmospheric sounding interferometer (IASI) because it provides the longest data record and the most comprehensive validations (47) among the satellite NH_3_ observations (70–72), as well as public access (73). The IASI v2.2R NH_3_ retrieval product data (2008 to 2017) are obtained from the MetOp-A (2008 to 2017) and -B (2013 to 2017) satellites (limited to cloud fraction ≤10%). The v2.2R retrieval is based on an artificial neural network for the IASI (74) with the European Centre for Medium‐Range Weather Forecasts Re‐Analysis (ERA) as its meteorological input (73). Only the morning orbits (~9:30 local solar time) are analyzed because of higher thermal contrast (sensitivity) versus the evening overpasses (75). A physical-based oversampling approach was applied to generate the 0.02 × 0.02° (~2 km) satellite NH_3_ maps (76, 77).

## Supplementary Material

Appendix 01 (PDF)

## Data Availability

The codes for the paper analyses are accessible at https://doi.org/10.5281/zenodo.10002062 (78). Data used within the manuscript come from previous publications referenced in the text.
