# Supplementary material for: Minimizing the impacts of the ammonia economy on the nitrogen cycle and climate
Source: Proc Natl Acad Sci U S A. 2023 Nov 6;120(46):e2311728120. doi: 10.1073/pnas.2311728120 (PMC10655559; doi:10.1073/pnas.2311728120)
Supplement: Supplementary file 1 — Appendix 01 (PDF) [file pnas.2311728120.sapp.pdf]

PNAS

Supporting Information for

Minimizing the Impacts of the Ammonia Economy on the Nitrogen Cycle and Climate.

Matteo B. Bertagni*, Robert H. Socolow, John Mark P. Martirez, Emily A. Carter, Chris Greig, Yiguang Ju, Tim Lieuwen, Michael E. Mueller, Sankaran Sundaresan, Rui Wang, Mark A. Zondlo, Amilcare Porporato*

* Matteo B. Bertagni (matteobb@princeton.edu), Amilcare Porporato (aporpora@princeton.edu)

This PDF file includes:

Supporting text (Sec. S1-S2)

Figures S1 to S2

Table S1

SI References

Sec S1. Environmental Impacts of Reactive Nitrogen Emissions

We review the environmental consequences of perturbing the nitrogen cycle (**Fig. S1**). We are facilitated in our goal by the extensive knowledge gained by the scientific community from the analysis of previous anthropogenic perturbations of the nitrogen cycle[1–3], mainly due to the production and use of nitrogen fertilizers in agriculture. The effects of each impact reviewed here may be compounded, giving rise to a cascade effect[4], whereby a reactive nitrogen species causing some adverse environmental impacts can be converted through natural processes into another reactive nitrogen species causing other impacts. For example, a nitrogen atom leaking away as NH_3 can contribute first to air pollution, then to water pollution in the form of NO_2^- ion, then to eutrophication of water bodies, and finally to global warming as N_2O. The cascade effect ends when the nitrogen atom is converted back from a reactive nitrogen species to the relatively unreactive N_2 molecule.

NH_3 is a corrosive gas. In ambient conditions, ammonia is a toxic gas with a pungent smell that favors leak detection. Ammonia toxicity is due to its alkaline properties, which make ammonia highly corrosive. NH_3 dissolves easily in water and air humidity, forming the alkaline ammonium ion (NH_4^+), which damages animal cells and causes caustic deposition. Depending on the exposure route, dose, and duration, ammonia's effects on humans can go from minor irritations of the eyes and respiratory tracts to major lung damage and death. Accidents from ammonia releases are not uncommon. Data based on 7619 US facilities with reportable ammonia inventories show that more than 1200 accidents occurred between 1994 and 2013, mainly due to equipment failure[5]. Eighteen of these accidents have caused human fatalities[5].

Air pollution due to NH_3 and NO_x emissions. Atmospheric NO_x (NO and NO_2) are highly reactive and control the tropospheric ozone production, a harmful air pollutant and a potent greenhouse gas. NO_x and NH_3 emissions are precursors to the formation and composition of unhealthy particulate matter (PM2.5), and ammoniated salts compose around one-third to one-half of PM2.5 in most areas[6]. These particles penetrate the lungs, damage the respiratory systems[7], and significantly contribute to increased mortality and morbidity[8–10]. Over 17,000 excess deaths annually are currently attributed to ammonia emissions in the United States alone[10]. Despite NO_x and NH_3 making similar contributions to particulate formation, visibility degradation, and water pollution following deposition, extensive regulations to control emissions are common worldwide only for NO_x and not NH_3[11].

Alteration of rain and surface water pH. NO_x and NH_3 can react with water directly in the atmosphere (in air humidity or rainwater) or surface water bodies after dry deposition. The products of the reactions are nitric acid (HNO_3) and the ammonium ion (NH_4^+), respectively, which are both highly corrosive. These products can also lead to significant changes in the water chemistry conditions. Nitric acid is responsible for water acidification (drop in pH), while ammonia is for water alkalinization (rise in pH). This has multiple consequences for the biosphere, human infrastructures, and other biogeochemical cycles. The lives of most aquatic organisms rely on a specific range of pH values. The consequences of a severe pH change on the

aquatic ecosystem can be disruptive, especially in the case of ammonia, which is also toxic to fish in very low concentrations. An example is an accident in 2004 in Kansas (USA), where ammonia spilled into a creek and killed around 25000 fish, including some threatened species[12]. Similarly, plants in forests and croplands thrive in favorable conditions of soil water chemistry and can be negatively affected by acid rain[13]. Rainwater enriched with corrosive compounds also has well-documented adverse consequences for human infrastructures. Finally, altering the natural water pH and alkalinity also impacts the carbon cycle by affecting the amount of CO_2 dissolved in water[14].

Water Pollution. High concentrations of nitrogen in drinking water are a threat to human health. The World Health Organization (WHO) standard for NO_3^- in drinking water is 50 mg/l for short-term exposure and 3 mg/l for chronic effects. These limits are often bypassed in rivers and groundwater due to the accumulation of nitrogen fertilizers[2]. Furthermore, nitrite ions (NO_2^-) threaten infant health by inactivating oxygen transport in the blood[3]. Because nitrate ions can form nitrite ions in the gastrointestinal tract, regulations usually deal with nitrate intake.

Eutrophication of aquatic ecosystems. Nitrogen is the limiting nutrient in many aquatic ecosystems, particularly in bays and estuaries. Deposition of reactive nitrogen can promote the growth of plants with fast nitrogen assimilation, ultimately leading to eutrophication, namely excessive plant growth. The consequences of eutrophication are disruptive and range from the bloom of harmful algae to the development of hypoxic conditions that can kill the whole aquatic biota, the so-called dead zones[15]. These problems are already widespread in estuaries and bays, where rivers discharge large amounts of nitrogen fertilizers from croplands. Estimates suggest that the number of coastal dead zones has doubled each decade since the 1960s[15]. A rise in reactive nitrogen deposition will exacerbate this trend.

Loss of Biodiversity. Anthropogenic deposition of reactive nitrogen has a fertilization effect that alters the pre-existing natural equilibrium of ecosystems. The consequences are multiple and still the argument of debate. In forest ecosystems, nitrogen additions can promote tree growth, increasing the biotic carbon stock (so-called nitrogen fertilization). However, nitrogen accumulation can also be a key driver of changes in species composition[13]. Species that use nitrogen more efficiently are favored at the expense of other species. The net effect can be a loss in biodiversity. For the temperate forests of northern Europe and North America, which have been the subjects of most scientific studies, nitrogen deposition has been suggested as one of the major risks to plant diversity degradation[13].

Global warming and depletion of stratospheric ozone by N_2O emissions. Nitrous oxide (N_2O) has a long average lifetime (~120 yr) in the atmosphere and a large capacity to absorb infrared radiation. Because of these reasons, N_2O has a heat-trapping capacity per kilogram that is 265 times higher than CO_2 over a 100-year time horizon. Once in the stratosphere, N_2O is depleted by ultraviolet radiation, forming NO_x as byproducts that act to deplete stratospheric ozone. Global N_2O emissions are estimated to be the current most critical ozone-depleting emissions. In addition to the direct N_2O emissions associated with inefficient

ammonia combustion, the *cascade* effect of reactive nitrogen might cause secondary emissions of N_2O following the deposition of ammonia and nitrates on land, where soil microorganisms responsible for the natural circulation of nitrogen in various forms convert some of the processed reactive nitrogen into N_2O.

Sec. S2. Future Prospects for Ammonia Technologies.

Given the environmental impacts of reactive nitrogen emissions, improvements in infrastructure and technologies to help mitigate them will be critical during the development of the ammonia economy. Materials and chemical engineering research could provide technological breakthroughs to alleviate some environmental concerns. Here we report some examples of strategies currently at low technological readiness levels, many not yet demonstrated at scale, that could improve the processes involved in the ammonia energy economy, potentially increasing overall chemical and energy efficiencies and reducing nitrogen emissions. Further literature reviews are also available on this topic (e.g., refs. [16,17]).

Ammonia synthesis. Strategies for greener ammonia production from N_2 with energy inputs from electricity-driven processes, including pulsed heating and quenching[18], plasma-assisted catalysis[19–21], and electrolysis[22] can be adopted. All of these methods are still in the proof-of-principle stage. For example, aqueous electrolysis results in very low yield, in many cases so low that detection and differentiation of the electrochemically generated NH_3 from N_2 vs. from N-containing impurities, including background NH_3, is a challenge[23]. The low yield is presumably due to low N_2 activation at room temperature. A solution to this seemingly insurmountable problem is to use mediators that can easily form nitrides from N_2; a prominent example is Li metal in an aprotic solvent[23]. The lithium nitride (Li3N) thus formed is exposed to water, forming NH_3. The LiOH formed after hydrolysis is reduced back to Li(s) via molten salt electrolysis[24]. The system thus cycles between NH_3 generation via hydrolysis of the mediator nitride, electrolytic reduction of the mediator oxide, and nitridation of the mediator metal catalyst. McEnaney et al. reported as much as 88.5 % current efficiency toward NH_3 synthesis under ambient pressures via this process[24]. As another example, pulsed, low-temperature plasma catalytic NH_3 production has achieved yields within a factor of four of laboratory-scale Haber-Bosch (for a fair comparison[21]).

Reactive nitrogen from the environment, in addition to atmospheric N_2, represents a potential source of nitrogen for NH_3 synthesis, which could partially offset the anthropogenic perturbation to the nitrogen cycle[25,26]. These reactive nitrogen sources include nitrates derived from agricultural runoff or wastewater treatment, where nitrate electrolysis can be combined with a water purification system[25,26]. In essence, the electrochemical process represents the reverse of the Ostwald process ($NH_3 + 2\ O_2 \longrightarrow H_2O + HNO_3$) and is theoretically more kinetically feasible at mild temperatures than breaking the strong nitrogen triple bond present in atmospheric N_2. Practically, several challenges must be addressed to enable the application of

such an ammonia synthesis, including ensuring an acceptable level of nitrate enrichment, achieving catalyst selectivity and durability, ensuring device portability, and improving the energy efficiency of the electrochemical process.

Non- or low-volatile forms of ammonia for storage. Unlike hydrogen and natural gas, NH_3 may be converted to denser and less volatile forms, partly due to its polar nature. The polarity and ability of NH_3 to form hydrogen bonds in fact enable its relatively safe storage as an aqueous solution; however, this method can't be used at scale and will incur a large energy penalty to separate NH_3 from water (e.g., alkalinization followed by distillation). Other clever alternatives can be and have been considered. Metal-ammines, e.g., magnesium or calcium chloride salts with molecular NH_3 as part of the crystal (akin to water crystallization as in hydrates), could be used for ammonia storage and transport, as they drastically reduce the vapor pressure of ammonia[27,28]. For example, $MgCl_2{\cdot}6NH_3$ has a vapor pressure of 0.0014 bar at 293 K, much lower than liquid NH_3 (8 bar at the same temperature) and far lower apparent toxicity level than gasoline (based on the ratios of their respective vapor pressure and immediately dangerous to life and health, IDLH, concentrations)[27]. Kilogram-scale production of this compound has already been demonstrated[29]. Another promising molecular conduit to stabilize ammonia is urea ($CO(NH_2)_2$). A solid at room temperature (with a melting point of 132.6 ^{o}C), urea can be generated from the reaction of CO_2 and NH_3. Urea is already co-produced in ammonia plants via this reaction at a rate of 150 Mt/yr (2010). The reaction of CO_2 and NH_3 is exothermic, forming ammonium carbamate ($NH_2CO_2NH_4$) that dehydrates to urea. Ammonia recovery could be facilitated by the thermal decomposition of urea, with isocyanic acid (HNCO) as a by-product. HNCO can hydrolyze to produce another NH_3 molecule and CO_2. Direct urea electrochemical synthesis from gaseous N_2 and CO_2 has been suggested recently[30], and other research to enable its production under mild conditions is also underway[31]. If the CO_2 used to produce urea is captured from biogenic emissions or directly from the air or bodies of water, then urea would be a net-zero carbon fuel, even though it releases CO_2 upon decomposition.

Ammonia cracking. Facilities dedicated to converting NH_3 back to H_2 could include new technologies that lower the reactor temperature to manage more easily ammonia recovery and recycling (chemical looping). Thermal decomposition methods that utilize other means of generating heat (other than fossil fuel combustion) or molecular activation have been explored and proposed, e.g., microwave, plasma, electric current, and solar concentrators[32]. For example, efforts to commercialize plasmonics (a kind of photocatalysis using metal nanoparticles) for such chemical processing are underway, e.g., Syzygy Plasmonics (https://plasmonics.tech/). In vehicles, direct ammonia fuel cells (DAFCs) use H_2 generated in-operando from NH_3, which is eventually oxidized to water at the anode. This DAFC approach is less likely to release N_2O and NO_x by-products when NH_3 is used as fuel in a fuel cell rather than combustion[33]. Moreover, exhaust processing units similar to catalytic converters in current motor vehicles could further mitigate reactive nitrogen emissions, converting them back to atmospheric N_2.

N_2O/NO_x capture and conversion. As stated above, oxygenated nitrogen that has escaped into the environment may be electrochemically reprocessed to re-generate NH_3. Capture and processing at point sources would of course be more efficient and less likely to affect human health and modify ecosystems. In selective catalytic reduction (SCR), NO_x species in flue gas, e.g., from coal-fired power plants, are reduced to N_2 and water with a reductant, such as NH_3 and urea, in the presence of a catalyst[34]. This is fortuitous in that such infrastructures already exist and the reductants of choice are already ammonia and urea. Furthermore, the typical catalyst used, namely, vanadium oxide on a titanium oxide substrate, is not particularly scarce, and SCR systems already achieve > 90% efficiency for direct NO_x removal. However, research in making such systems portable, resilient, and deployable in many other applications will be essential as ammonia use becomes more widespread. For example, finding materials that improve the lifetime and resistance to deactivation and fouling of SCR catalysts will be important.

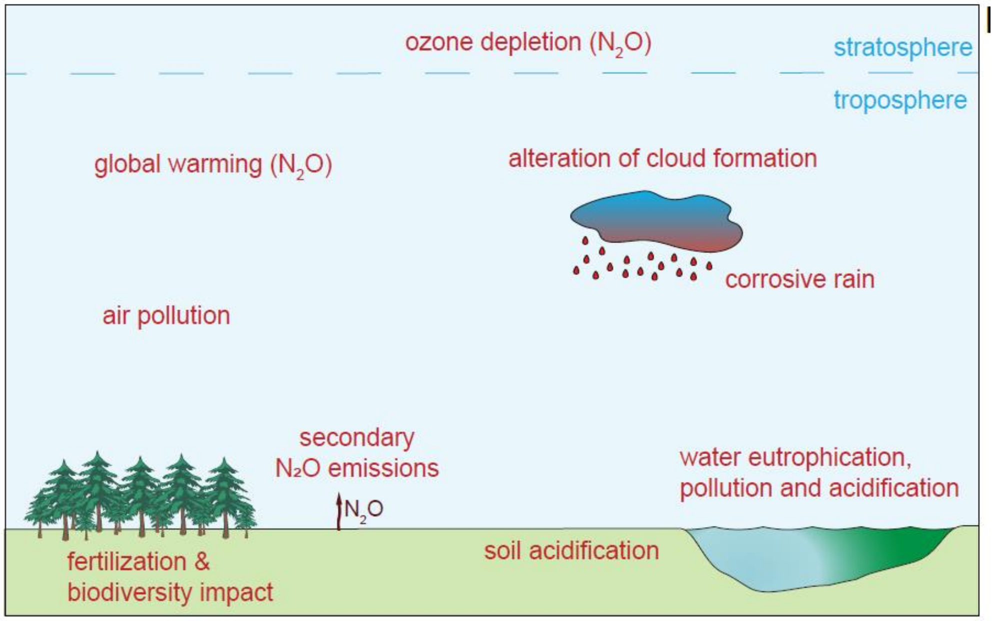

Figure S1. Environmental impacts of perturbing the nitrogen cycle.

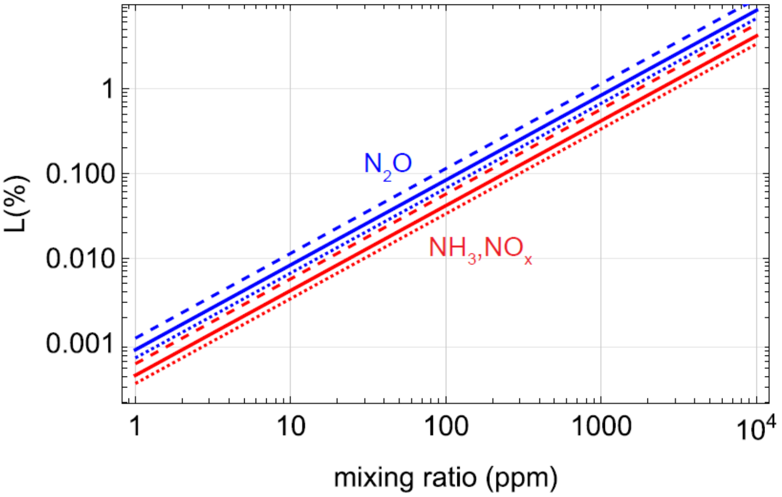

Figure S2. Loss rates (L in %) of nitrogen species as a function of the mixing ratio (ppm) in the exhaust for combustion of ammonia with air. Results from eq. **(6)** and **(7)**. Blue lines are for N_2O, and red lines are for NH_3 and NO_x. The equivalence ratio φ is 0.7 (dashed), 1 (continuous), 1.3 (dotted).

Fuel	Volumetric Energy density (MJ/l)	Gravimetric energy density (MJ/kg)	Flame velocity (m/s)	Flammability limits in the air (vol%)	Minimum ignition energy (mJ)
H_2	0.01<br>2.5[a]<br>8.5[b]	120	3.25	4 - 75	680
NH_3	0.013<br>~12[c]	19	0.067	15 - 28	~0.02
Gasoline iso-octane	31	44	0.41	0.6-8	~0.14

[a]H_2 compressed at 300 atm and standard temperature
[b]Liquid H_2 cooled at -253°C and atmospheric pressure
[c]Liquid NH_3 cooled at -33°C and standard pressure, or compressed at 10 atm and standard temperature

Table S1. Properties of H_2, NH_3, and gasoline fuels. Energy densities are defined with lower heating values (LHVs). If not specified, standard conditions apply. Data are from Ref.[35].

SI References

1. Fowler, D. *et al.* The global nitrogen cycle in the twenty-first century. *Philos. Trans. R. Soc. B Biol. Sci.* **368**, 20130164 (2013).
2. Erisman, J. W. *et al.* Consequences of human modification of the global nitrogen cycle. *Philos. Trans. R. Soc. B Biol. Sci.* **368**, 20130116 (2013).
3. Socolow, R. H. Nitrogen management and the future of food: Lessons from the management of energy and carbon. *Proc. Natl. Acad. Sci.* **96**, 6001–6008 (1999).
4. Galloway, J. N. *et al.* The Nitrogen Cascade. *BioScience* **53**, 341–356 (2003).
5. Anderson, M. K. Ammonia safety, a global perspective. 69 (2017).
6. Zhang, Q. *et al.* Ubiquity and dominance of oxygenated species in organic aerosols in anthropogenically-influenced Northern Hemisphere midlatitudes. *Geophys. Res. Lett.* **34**, (2007).
7. Wilson, R. & Spengler, J. D. Particles in our air: concentrations and health effects. (1996).
8. Vohra, K. *et al.* Rapid rise in premature mortality due to anthropogenic air pollution in fast-growing tropical cities from 2005 to 2018. *Sci. Adv.* **8**, eabm4435 (2022).
9. Gu, B. *et al.* Abating ammonia is more cost-effective than nitrogen oxides for mitigating PM2.5 air pollution. *Science* **374**, 758–762 (2021).
10. Thakrar, S. K. *et al.* Reducing Mortality from Air Pollution in the United States by Targeting Specific Emission Sources. *Environ. Sci. Technol. Lett.* **7**, 639–645 (2020).
11. Behera, S. N., Sharma, M., Aneja, V. P. & Balasubramanian, R. Ammonia in the atmosphere: a review on emission sources, atmospheric chemistry and deposition on terrestrial bodies. *Environ. Sci. Pollut. Res. Int.* **20**, 8092–8131 (2013).
12. Rosenker, M. V. *Report of Kansas Ammonia Accident*. (2004).
13. Bobbink, R. *et al.* Global assessment of nitrogen deposition effects on terrestrial plant diversity: a synthesis. *Ecol. Appl.* **20**, 30–59 (2010).
14. Bertagni, M. B. & Porporato, A. The Carbon-Capture Efficiency of Natural Water Alkalinization: Implications For Enhanced weathering. *Sci. Total Environ.* **838**, 156524 (2022).
15. Diaz, R. J. & Rosenberg, R. Spreading Dead Zones and Consequences for Marine Ecosystems. *Science* **321**, 926–929 (2008).
16. Morlanés, N. *et al.* A technological roadmap to the ammonia energy economy: Current state and missing technologies. *Chem. Eng. J.* **408**, 127310 (2021).
17. MacFarlane, D. R. *et al.* A Roadmap to the Ammonia Economy. *Joule* **4**, 1186–1205 (2020).
18. Dong, Q. *et al.* Programmable heating and quenching for efficient thermochemical synthesis. *Nature* **605**, 470–476 (2022).
19. Chen, Z., Jaiswal, S., Diallo, A., Sundaresan, S. & Koel, B. E. Effect of Porous Catalyst Support on Plasma-Assisted Catalysis for Ammonia Synthesis. *J. Phys. Chem. A* **126**, 8741–8752 (2022).
20. Wang, Y. *et al.* Plasma-Enhanced Catalytic Synthesis of Ammonia over a Ni/Al2O3 Catalyst at Near-Room Temperature: Insights into the Importance of the Catalyst Surface on the Reaction Mechanism. *ACS Catal.* **9**, 10780–10793 (2019).
21. Kim, H.-H., Teramoto, Y., Ogata, A., Takagi, H. & Nanba, T. Atmospheric-pressure nonthermal plasma synthesis of ammonia over ruthenium catalysts. *Plasma Process. Polym.* **14**, 1600157 (2017).
22. Yang, B., Ding, W., Zhang, H. & Zhang, S. Recent progress in electrochemical synthesis of ammonia from nitrogen: strategies to improve the catalytic activity and selectivity. *Energy Environ. Sci.* **14**, 672–687 (2021).
23. Qing, G. *et al.* Recent Advances and Challenges of Electrocatalytic N2 Reduction to Ammonia. *Chem. Rev.* **120**, 5437–5516 (2020).
24. McEnaney, J. M. *et al.* Ammonia synthesis from N2 and H2O using a lithium cycling electrification strategy at atmospheric pressure. *Energy Environ. Sci.* **10**, 1621–1630 (2017).
25. van Langevelde, P. H., Katsounaros, I. & Koper, M. T. M. Electrocatalytic Nitrate Reduction for Sustainable Ammonia Production. *Joule* **5**, 290–294 (2021).
26. Anastasiadou, D., van Beek, Y., Hensen, E. J. M. & Costa Figueiredo, M. Ammonia electrocatalytic synthesis from nitrate. *Electrochem. Sci. Adv.* **n/a**, e2100220.
27. Klerke, A., Christensen, C. H., Nørskov, J. K. & Vegge, T. Ammonia for hydrogen storage: challenges and opportunities. *J. Mater. Chem.* **18**, 2304–2310 (2008).
28. Sørensen, R. Z. *et al.* Indirect, Reversible High-Density Hydrogen Storage in Compact Metal Ammine Salts. *J. Am. Chem. Soc.* **130**, 8660–8668 (2008).
29. Zhu, H., Gu, X., Yao, K., Gao, L. & Chen, J. Large-Scale Synthesis of MgCl2·6NH3 as an Ammonia Storage Material. *Ind. Eng. Chem. Res.* **48**, 5317–5320 (2009).
30. Chen, C. *et al.* Coupling N2 and CO2 in H2O to synthesize urea under ambient conditions. *Nat. Chem.* **12**, 717–724 (2020).
31. Yuan, M. *et al.* Unveiling Electrochemical Urea Synthesis by Co-Activation of CO2 and N2 with Mott–Schottky Heterostructure Catalysts. *Angew. Chem.* **133**, 11005–11013 (2021).

32. Lucentini, I., Garcia, X., Vendrell, X. & Llorca, J. Review of the Decomposition of Ammonia to Generate Hydrogen. *Ind. Eng. Chem. Res.* **60**, 18560–18611 (2021).
33. Jeerh, G., Zhang, M. & Tao, S. Recent progress in ammonia fuel cells and their potential applications. *J. Mater. Chem. A* **9**, 727–752 (2021).
34. Romero, C. E. & Wang, X. Chapter Three - Key technologies for ultra-low emissions from coal-fired power plants. in *Advances in Ultra-Low Emission Control Technologies for Coal-Fired Power Plants* (eds. Zhang, Y., Wang, T., Pan, W.-P. & Romero, C. E.) 39–79 (Woodhead Publishing, 2019). doi:10.1016/B978-0-08-102418-8.00003-6.
35. Tornatore, C., Marchitto, L., Sabia, P. & De Joannon, M. Ammonia as Green Fuel in Internal Combustion Engines: State-of-the-Art and Future Perspectives. *Front. Mech. Eng.* **8**, (2022).
